# Red Blood Cells Protect Albumin from Cigarette Smoke–Induced Oxidation

**DOI:** 10.1371/journal.pone.0029930

**Published:** 2012-01-04

**Authors:** Graziano Colombo, Ranieri Rossi, Nicoletta Gagliano, Nicola Portinaro, Marco Clerici, Andrea Annibal, Daniela Giustarini, Roberto Colombo, Aldo Milzani, Isabella Dalle-Donne

**Affiliations:** 1 Department of Biology, Università degli Studi di Milano, Milan, Italy; 2 Department of Evolutionary Biology, University of Siena, Siena, Italy; 3 Department of Human Morphology and Biomedical Sciences “Città Studi”, Università degli Studi di Milano, Milan, Italy; 4 Department of Translational Medicine, Clinica Ortopedica e Traumatologica, Istituto Clinico Humanitas and Università degli Studi di Milano, Rozzano, Milan, Italy; Semmelweis University, Hungary

## Abstract

Different studies reported the presence of oxidized (carbonylated) albumin in the extravascular pool, but not in the intravascular one of cigarette smokers. In this study we attempted to explain this apparent discrepancy exposing human serum albumin (HSA) to aqueous cigarette smoke extract (CSE). CSE induces HSA carbonylation and oxidation of the HSA Cys34 sulfhydryl group. An antioxidant action of glutathione, cysteine, and its synthetic derivative *N*-acetylcysteine was observed only at supra-physiological concentrations, suggesting that physiological (plasma) concentrations of glutathione and cysteine in the low micromolar range are ineffective in preventing cigarette smoke–induced oxidation of HSA. Differently, human erythrocytes resulted to be protective towards CSE-induced oxidation (carbonylation and thiol oxidation) of both HSA and total human plasma proteins.

## Introduction

Cigarette smoke (CS), an etiological factor for the development of many tobacco-related diseases [1]–[3], is a complex mixture containing more than 7000 different constituents, including reactive oxygen and nitrogen species (ROS and RNS) [4]. Further ROS/RNS production mediated through inflammatory processes may exacerbate those produced through direct exposure [5]. One pathway that may contribute to the untoward health effects of CS is the systemic oxidants/antioxidants imbalance as reflected by increased levels of products of oxidative stress and depleted levels of antioxidants in plasma of smokers [6].

Oxidative damage induced by CS is caused by some water-soluble oxidants that may undergo circulation through the body fluids and produce sustained oxidative damage in different organ systems [5]–[7]. Human plasma antioxidants include the low molecular mass aminothiols glutathione (GSH) and cysteine (Cys-SH), which occur in the micromolar range [8]. Their concentrations in plasma are decreased in association with cigarette smoking [9], [10].

Protein thiol groups (PSH) are also essential in conferring protection against oxidative stress [8], [11]–[13]. In plasma, concentration of PSH is mostly due to the single free thiol (Cys34) of albumin, a single-chain polypeptide with 17 disulfide bonds, which reaches concentrations of more than 0.6 mM (mean ∼43 mg·ml^−1^, range 35–52 mg·ml^−1^) in healthy humans [14], accounting for ∼60% of total protein in the plasma of healthy people [15]. As a consequence, the Cys34 sulfhydryl group of human serum albumin (HSA) represents by far the largest fraction (∼80%) of all free thiols in plasma, thus conferring a major role in serum antioxidant capacity to HSA, which therefore represents a quantitatively important redox buffer as well as the main scavenger of electrophiles of the blood [16]–[19].

Increased levels of variously oxidized proteins have been found in blood and/or plasma of smokers, but none of those studies reported the occurrence of oxidized albumin [20]–[23]. Differently, other studies showed that the major oxidized (carbonylated) protein in the bronchoalveolar lavage fluid in smokers with a long term history of smoking is albumin [24], [25]. A recent study determined that human lung parenchymal tissue from COPD patients who were current smokers contained lower levels of total HSA, but had proportionally greater levels of oxidized (carbonylated) HSA, compared to patients with normal lung function. Lung tissue from current smokers was also found to contain lower levels of HSA, which was highly carbonylated compared to lung tissue from ex-smokers and non-smokers [26].

We previously showed α,β-unsaturated aldehyde-induced carbonylation in HSA exposed to whole-phase cigarette smoke extract (CSE), a widely used model system for studying in vitro effects of CS [27]–[30] and identified the amino acids forming covalent adducts with acrolein and crotonaldehyde [30]. We investigated here the ability of some plasma low molecular mass antioxidants (i.e., GSH and Cys-SH) and the synthetic cysteine derivative *N*-acetylcysteine (NAC) to protect HSA from CSE-induced oxidation. Furthermore, considering that carbonylated HSA has been found in fluids and tissues of smokers other than blood or plasma and also considering the preponderance of GSH within erythrocytes (which contain ∼3 mM GSH, i.e., one order of magnitude higher than that present in plasma) besides several other antioxidants (amongst which peroxiredoxins are predominant; [31]), we asked whether red blood cells (RBCs) were protective towards CSE-induced oxidation of both purified HSA and human plasma proteins as a whole.

## Materials and Methods

### Ethics Statement

Human blood samples were obtained from healthy donors that voluntarily went to the Analysis Laboratory for a routinary blood check-up, after informed verbal consent. The verbal consent was considered to be sufficient because the samples were handled anonymously and were used only to isolate erythrocytes and plasma proteins. The procedure was approved by the ethics committee of the University of Milan.

### Materials

Delipidized crystalline HSA (∼99% agarose gel electrophoresis), 5,5′-dithiobis(2-nitrobenzoic acid) (DTNB), *N*-ethylmaleimide (NEM) and 2,4-dinitrophenylhydrazine (DNPH) were purchased from Sigma-Aldrich (Milan, Italy). ECL Plus Western blotting detection reagents were obtained from GE Healthcare (Milan, Italy). EZ-Link Biotin-HPDP (*N*-(6-(Biotinamido)hexyl)-3′-(2′-pyridyldithio)-propionamide) was obtained from Euroclone (Pero, Milan, Italy). All other reagents were of analytical grade (Sigma-Aldrich, Milan, Italy). The slot-blotter (Bio-Dot SF apparatus) was obtained from Bio-Rad Laboratories (Hercules, CA, USA). Research-grade cigarettes (3R4F) were purchased from the College of Agriculture c/o Kentucky Tobacco Research & Development Center, University of Kentucky (USA).

### Preparation of human mercaptalbumin (HSA-SH)

Delipidized HSA (12 mg/ml, 0.18 mM) was quantitatively converted to mercaptalbumin (HSA-SH), in which the single thiol of HSA is completely reduced, by treatment with 1.5 mM dithiotreitol (DTT) in 50 mM potassium phosphate buffer (PBS), pH 7.4, for 15 min, at room temperature. The excess of DTT was then removed by exhaustive dialysis against 50 mM PBS, pH 7.4.

### Preparation of whole cigarette smoke extract (CSE)

Whole phase CSE from Kentucky 3RF4 reference cigarettes was prepared as previously described [30]. Mainstream smoke from one cigarette (10 puffs) was allowed to dissolve (for 10 s each puff) in one ml of 50 mM PBS, pH 7.4. The resultant dark yellow solution was defined as 100% whole phase CSE and was filtered through a 0.22 µm Millipore filter (Bedford, MA, USA) to remove bacteria and large particles. The pH of the whole phase CSE was adjusted to 7.4 by addition of 2 M sodium hydroxide solution**.** To ensure standardization between experiments and batches of CSE, CSE preparations were uniformed by measurement of absorbance at 340 nm. CSE was freshly prepared immediately before use for each experiment and diluted to appropriate concentration with 50 mM PBS, pH 7.4.

### Blood collection and isolation of erythrocytes and plasma proteins

Human blood was obtained in the morning after 10–12 h of starving from the antecubital vein. K_3_EDTA was used as an anticoagulant. Blood samples were partitioned into two groups: one group was used for collecting RBCs and the other one was used for collecting plasma proteins. RBCs were collected by centrifugation at 10,000 *g* for 20 s and washed three times with 200 mM Na^+^/K^+^ phosphate buffered saline (PBS, pH 7.4)/NaCl 0.9% (w/v), 1∶9 v/v ratio (PBS/NaCl), containing 5 mM glucose. The washed RBCs were suspended in PBS/NaCl containing 5 mM glucose to a hematocrit value of 20%.

Plasma proteins were obtained by blood centrifugation at 1000 *g* for 10 min, at 4°C.

### Exposure of HSA-SH to CSE

HSA-SH (molecular mass, 66.438 kDa) concentration was determined by measuring the absorbance at 280 nm, using an extinction coefficient of 39,800 M^−1^cm^−1^. In a typical experiment, HSA-SH was diluted with 50 mM PBS, pH 7.4, to a final concentration of 4 mg/ml (60 µM) and treated for 60 min, at 37°C, with various concentrations (1%, 4%, and 16%, v/v) of aqueous CSE, with gentle rotary shaking. For experiments performed with RBCs, HSA-SH solutions were exposed to 1–16% (v/v) CSE for 60 min, at 37°C, in the absence or presence of 2.5% (v/v) or 5% (v/v) erythrocytes (i.e., equal to about one tenth of the mean human hematocrit value as in our experiments HSA concentration too is equal to about one tenth of the mean plasma HSA concentration) in 200 mM PBS/NaCl (see above), containing 5 mM glucose. The removal of CSE was accomplished by acetone precipitation: protein samples were mixed with three volumes of 100% acetone, allowed to precipitate for 30 min at −20°C and then centrifuged at 10,000 *g* for 10 min, at 4°C. Pellet were washed with 70% acetone and centrifuged at 10,000 *g* for 10 min, at 4°C. Finally, dried pellets were re-suspended in 50 mM PBS, pH 7.4. All reported experiments were carried on in CSE-free buffer.

### Exposure of human plasma proteins to CSE

Plasma protein concentration was determined by Bradford assay. Experiments with plasma proteins and/or RBCs were performed at 37°C, at the protein concentration of 4 mg/ml (i.e., equal to one tenth of the mean HSA concentration) and with washed RBCs re-suspended in PBS/NaCl containing 5 mM glucose at a hematocrit of either 5% (v/v; i.e., equal to about one tenth of the mean human hematocrit value) or 2.5% (v/v).

Plasma proteins, in the absence or presence of 2.5% or 5% (v/v) RBCs, were exposed to various concentrations (1%, 4%, and 16%, v/v) of CSE PBS/NaCl/glucose, at 37°C, in a water bath. After a 60-min incubation, RBCs were separated by centrifugation at 10,000 g for 20 s and supernatant with plasma proteins collected in a new microcentrifuge tube. The removal of CSE was accomplished by acetone precipitation as described above for albumin exposure to CSE. Depending on experimental analysis, protein pellet was alternatively resuspended in either PBS/NaCl or PBS/NaCl containing 0.4 mM biotin–HPDP for carbonyl analysis or free –SH determination, respectively.

### Determination of HSA Cys34 free sulfhydryl group by the Ellman assay

Cysteine residues were quantified by the Ellman assay [32]. Following exhaustive dialysis against 50 mM PBS, pH 7.4, protein samples (600 µg in 950 µl) were added with 50 µl of 3 mM DTNB (prepared in 0.05 M phosphate buffer, pH 7.4) and incubated for 15 min at 25°C. The number of cysteines was determined by measuring the increase in absorbance caused by the released TNB anion upon reaction of a thiol with DTNB at 412 nm and using a molar absorption coefficient of 14.15 mM^−1^ cm^−1^ [33]. From the increased absorbance in protein samples, the molar concentration of Cys34 thiol was calculated from the molar absorbance of the TNB anion.

### Determination of HSA Cys34 and plasma protein free sulfhydryl group by biotin–HPDP binding and Western blot analysis

After treatment with various concentrations of CSE and CSE removal by acetone precipitation as described above, pellets of HSA samples (400 µg) or plasma proteins (400 µg) were resuspendend, respectively, in 50 mM PBS, pH 7.4, or PBS/NaCl containing 0.4 mM biotin–HPDP (stock solution 4 mM in 90% dimethyl sulfoxide and 10% dimethylformamide) and mixed by gently vortexing. The biotinylation reaction was carried out at room temperature in the dark for 60 min, mixing by vortex every 15 min from the start of incubation. To remove biotin-HPDP excess, biotin-HPDP-labeled protein samples were precipitated with acetone as in the previous step and resuspendend with an equal volume of 2× non-reducing SDS-PAGE sample buffer. Samples were then run on SDS–PAGE on Tris–HCl 10% resolving gels and electroblotted on to Immobilon P polyvinylidene difluoride (PVDF; Sigma-Aldrich, Milan, Italy) membrane or stored at −20°C for later use. To detect HSA sulfhydryl groups/Cys34 labeled with biotin–HPDP, membranes were blocked for 1 h in 5% (w/v) non-fat dry milk in PBST [10 mM Na-phosphate, pH 7.2, 0.9% (w/v) NaCl, 0.1% (v/v) Tween 20] and probed with horseradish peroxidase (HRP)-conjugated streptavidin (1∶5000 dilution) for 2 h in 5% (w/v) non-fat dry milk in PBST. After washing in PBST, immunoreactive bands were detected by using enhanced chemiluminescence. Protein bands were then visualized by washing the membrane extensively in PBS and then staining with Amido black.

### Determination of HSA Cys34 free sulfhydryl group by biotin–HPDP binding and slot blot analysis

HSA samples were treated with CSE as described above. Protein pellets were resuspendend in 50 mM PBS, pH 7.4 and each sample type was incubated with DTT (0–2 mM) for 15 min at room temperature. A series of control and CSE-treated HSA samples was added with 20 mM of the thiol alkylating agent NEM and incubated for 30 min at 50°C before DTT addition. Protein precipitation and the biotinylation reaction were induced and carried out, respectively, as described above. An Immobilon P PVDF membrane was prepared by wetting it with 100% methanol for 5 min and then soaking it in a 100% Tris-buffered saline solution (TBS) (20 mM Tris-base, pH 7.5, 500 mM NaCl) for 15 min. Protein pellets were resuspended in PBS and 0.6 ml of TBS-diluted albumin solutions (3 µg total protein) was applied to each slot. After three washes with TBS, the membrane was probed with HRP-conjugated streptavidin and developed by using enhanced chemiluminescence as described above for the Western blot analysis.

### Incubation with GSH, Cys-SH or *N*-acetylcysteine (NAC) before HSA-SH exposure to CSE

In order to evaluate the possible effectiveness of plasma low molecular mass aminothiols in preventing CSE-induced oxidation of albumin Cys34, HSA-SH samples were pre-incubated for 30 min with increasing concentrations of GSH, i.e., 0.3, 3 or 30 µM, corresponding to [GSH]/[HSA] molar ratios of 0.005 (1×, mean physiological molar ratio), 0.05 (10×) or 0.5 (100×), respectively, or Cys-SH, i.e., 1, 10 or 100 µM, corresponding to [Cys-SH]/[HSA] molar ratios of 0.016 (1×, mean physiological molar ratio), 0.16 (10×) or 1.6 (100×), respectively, before exposure to 1–16% (v/v) CSE. Parallel samples were prepared in which GSH or Cys-SH were added just before exposure to 1–16% (v/v) CSE.

Another set of HSA-SH samples were pre-incubated for 30 min with 1, 10 or 100 µM NAC before exposure to 1–16% (v/v) CSE. Parallel samples were prepared in which NAC was added just before exposure to 1–16% (v/v) CSE.

### Reduction of reversible sulfhydryl modifications

After treatment with CSE (see above), reduction of HSA reversible sulfhydryl modifications was accomplished by treatment with different concentrations of DTT in 50 mM PBS, pH 7.4, for 15 min at room temperature. Alternatively, HSA reversible sulfhydryl modifications were reduced through prolonged (two days) dialysis against a 100-fold volume of 50 mM PBS, pH 7.4, added with 5 mM GSH, at 4°C. Buffer changes were accomplished every 24 h.

### Spectrophotometric determination of protein carbonylation

Carbonyl groups formed on human plasma proteins were quantified by adding an equal volume of 10 mM 2,4-dinitrophenylhydrazine (DNPH) in 2 M HCl to the different plasma solutions containing 2 mg of total proteins; these were allowed to stand in the dark at room temperature for 1 h, with vortexing every 10 min. Samples were precipitated with TCA (20% final concentration) and centrifuged at 13,000 *g* in a tabletop microcentrifuge for 5 min, at room temperature. The supernatants were discarded and the protein pellets were washed once more with 20% TCA and then washed at least three times with 1 ml portions of ethanol/ethylacetate (1∶1) to remove any free DNPH. The protein samples were resuspended in 1 ml of 6 M guanidine hydrochloride (dissolved in 20 mM phosphate buffer, pH 2.3) at 37°C, for 15 min, with vortex mixing. Carbonyl contents were determined from the absorbance at 366 nm using a molar absorption coefficient of 22,000 M^−1^ cm^−1^ [34].

### Densitometric analysis

Densitometric analysis was performed after scanning the chemiluminescence films using Image J 1.40d (National Institutes of Health, Bethesda, MD, USA).

## Results

### Effect of CSE on HSA Cys34 free sulfhydryl group and on the HSA structure as a whole

**Figure 1:**
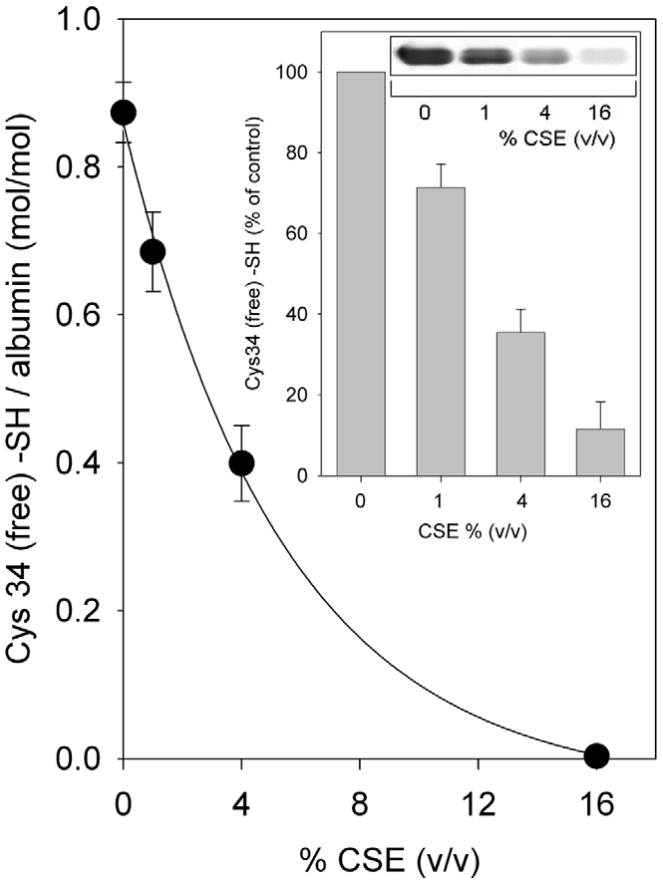
Effect of CSE on HSA Cys34 free sulfhydryl group as determined by the Ellman assay. HSA-SH solutions (60 µM) were treated for 60 min with vehicle (control) or 1%, 4% and 16% (v/v) CSE and then exhaustively dialyzed. The concentration of Cys34 sulfhydryl groups in HSA samples was determined by the Ellman assay at 412 nm as described under Materials and methods. Data are presented as the mean ± SD of three independent measurements. Inset: Effect of CSE on HSA Cys34 free sulfhydryl group as determined by biotin-HPDP binding and Western blot analysis. HSA-SH solutions (60 µM) were treated for 60 min with vehicle (control) or 1%, 4% and 16% CSE, exhaustively dialyzed and then labeled at Cys34 with biotin-HPDP as described under Materials and methods. Proteins (10 µg/lane) were separated by SDS-PAGE and biotin-HPDP binding was detected by Western blot analysis using streptavidin-HRP as described under Materials and methods (immunoblot inset). Amido Black staining of the same PVDF membrane showed equal protein loading and transfer (not shown). Immunoblot shown is representative of three independent determinations. Bar-graph inset shows densitometric analysis of biotin-HPDP incorporation. Data are presented as the mean ± SD of three independent determinations.

When HSA-SH solutions were incubated with increasing concentrations (0–16%, v/v) of CSE, the number of sulfhydryl groups, as determined by reaction with DTNB, progressively decreased from 0.90±0.04 mol -SH/mol albumin to 0 mol -SH/mol albumin (Fig. 1). Cys34 thiol modification was further established in a complementary experiment by using one of the biotin-based tagging techniques, which have been applied with success to monitor the posttranslational modification of protein thiols by ROS and electrophilic compounds [35]–[37]. The biotin tag can be detected at a level of sensitivity in the picomole range using immunoblotting with HRP-conjugated streptavidin [37]. In our case, the loss of the biotin signal is proportional to the degree of protein thiol modification. The results of the biotin-based tagging experiment, after separation by non-reducing SDS-PAGE and Western blotting, are presented in Fig. 1, inset. The inset upper panel depicts results obtained when the membrane was assayed for free sulfhydryl groups using immunochemical detection of biotin-HPDP with HRP-conjugated streptavidin. The albumin exposed to CSE clearly exhibited a decrease in the Cys34 sulfhydryl group relative to HSA-SH. Albumin staining with Amido black performed on the same membrane evidenced that total protein loaded in each lane was the same (not shown). The inset bar graph shows densitometric analysis of biotin-HPDP incorporation.

Exposure of HSA to 1–16% (v/v) CSE did not result in large protein damage such as fragmentation or aggregation due to intermolecular dityrosine, as assessed by reducing SDS-PAGE (not shown). Protein analysis by SDS-PAGE under non-reducing conditions demonstrated that no intermolecular disulfide linkage was elicited in HSA by CSE exposure (not shown).

### Reversibility of Cys34 oxidation

**Figure 2:**
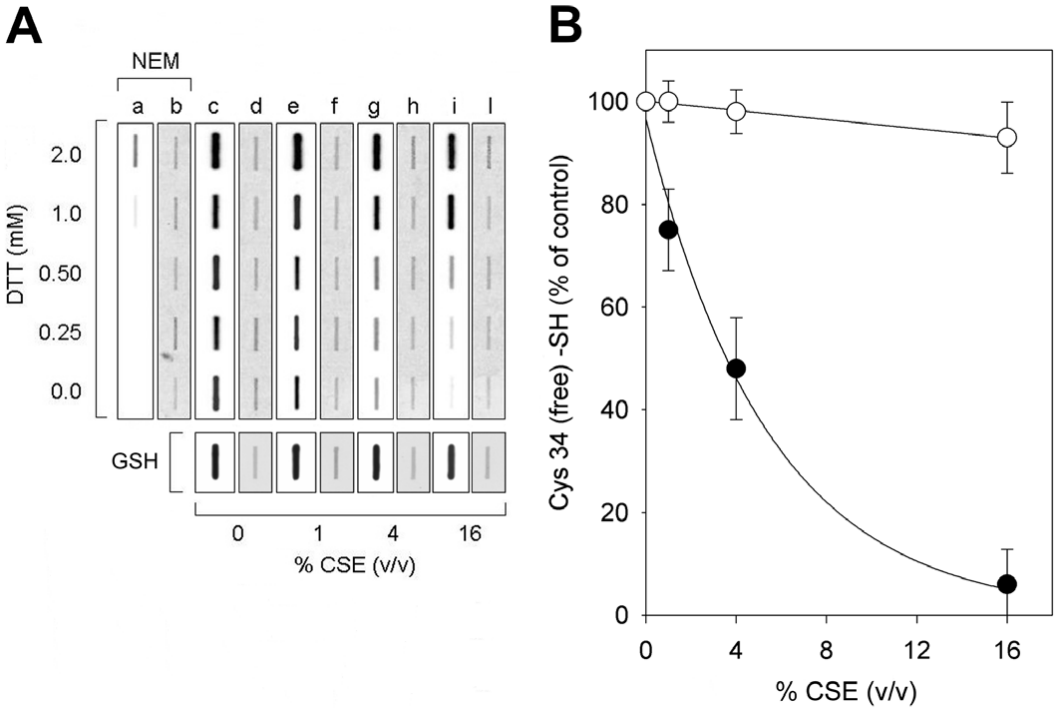
Reversibility of Cys34 oxidation as determined by biotin–HPDP binding and slot blot analysis. (**A**) HSA samples (60 µM) were treated with CSE and then incubated for 15 min with different concentrations of DTT as described under Materials and methods. The biotinylation reaction was carried out as described under Materials and methods. A series of control and CSE-treated HSA samples was added with 20 mM NEM and incubated for 30 min at 50°C before DTT addition (strips marked with NEM). In another series of control and CSE-treated HSA samples, reduction of HSA sulfhydryl modifications was obtained through a prolonged dialysis against 50 mM PBS, pH 7.4, added with 5 mM GSH (strips marked with GSH). After protein precipitation and resuspension in TBS, diluted protein solutions (3 µg total protein) were applied to each slot and the membrane was probed with HRP-conjugated streptavidin and developed by using enhanced chemiluminescence (strips a, c, e, g, i) as described under Materials and methods. Strips marked with b, d, f, h, l show the corresponding duplicate slot-blot stained for proteins with Amido black. (**B**) Graph shows densitometric analysis of biotin-HPDP incorporation in HSA samples treated with CSE without further DTT, NEM or dialysis against GSH (filled circles) and in HSA samples treated with CSE and then dialyzed against GSH (open circles). Data are presented as the mean ± SD of three independent determinations.

Oxidation of HSA Cys34 thiol is partially reversible in the presence of 0.25 and 0.5 mM DTT and almost completely reversible in the presence of 1–2 mM DTT. Fig. 2A shows the detection of the Cys34 free sulfhydryl group in both control and CSE-exposed HSA, in the presence of increasing concentrations of DTT, by means of biotin-HPDP binding and slot-blot analysis with streptavidin-HRP (strips a, c, e, g, i). The basal level of the free sulfhydryl group at Cys34 in HSA-SH was easily detectable (Fig. 2A, strip c, absence of DTT), whereas the thiol of Cys34 decreased after HSA exposure to 1–16% (v/v) CSE (Fig. 2A, strips e, g, i, absence of DTT). Treatment of parallel samples with the reducing agent DTT partially (0.25–0.5 mM DTT) or completely (1–2 mM DTT) abolished Cys34 oxidation in HSA exposed to 1–16% CSE (Fig. 2A, strips c, e, g, i), as judged by the appearance of the chemiluminescence signal related to biotinylation of the thiol of Cys34, suggesting a mainly reversible oxidation of Cys34 induced by 1–16% CSE. Membrane strips marked with NEM refer to HSA samples in which the Cys34 sulfhydryl group was alkylated with 20 mM NEM at 50°C before DTT addition. In these samples, no biotinylation signal was expected as the only HSA free sulfhydryl group was covalently blocked with NEM. Detection of biotin-HPDP binding in the sample treated with 2 mM DTT, and to a much lesser extent in that treated with 1 mM DTT, suggests that the highest DTT concentrations caused cleavage of one or more out of the 17 albumin disulfide bond(s) and, consequently, exposure of additional –SH groups (Fig. 2A, strip a). Such a drastic reductive effect of 1–2 mM DTT obviously occurred in CSE-treated HSA samples too, therefore in such samples a small (2 mM DTT) or minimal (1 mM DTT) part of the biotinylation signal is due to exposure of additional –SH groups because of the breaking of one or more HSA disulfide bond(s). Strips marked with b, d, f, h, and l depict the same slot blot membrane strips after Amido black staining.

In order to avoid any undesired and uncontrolled breaking of intramolecular disulfides within the HSA molecule, we performed an additional experiment reducing HSA sulfhydryl modifications through a prolonged (two days) dialysis against a 100-fold volume of 50 mM PBS, pH 7.4, added with 5 mM GSH, showing that GSH is able to almost completely revert Cys34 oxidations induced by 1–16% (v/v) CSE (Fig. 2A, strips c, e, g, l plus GSH) except for a minimal amount (about 5–6%) of, likely, irreversible oxidations occurring in HSA samples treated with 16% CSE (Fig. 2B). Preliminary mass spectrometry data reveal that about 6% of Cys34 occurs as sulfinic acid in HSA samples exposed to 16% CSE (our laboratory, unpublished data). Therefore, we can guess that the great majority of oxidative modifications occurring at the Cys34 –SH group are reversible ones.

### Effects of physiological and supra-physiological concentrations of GSH and Cys-SH on CSE-induced Cys34 oxidation

**Figure 3:**
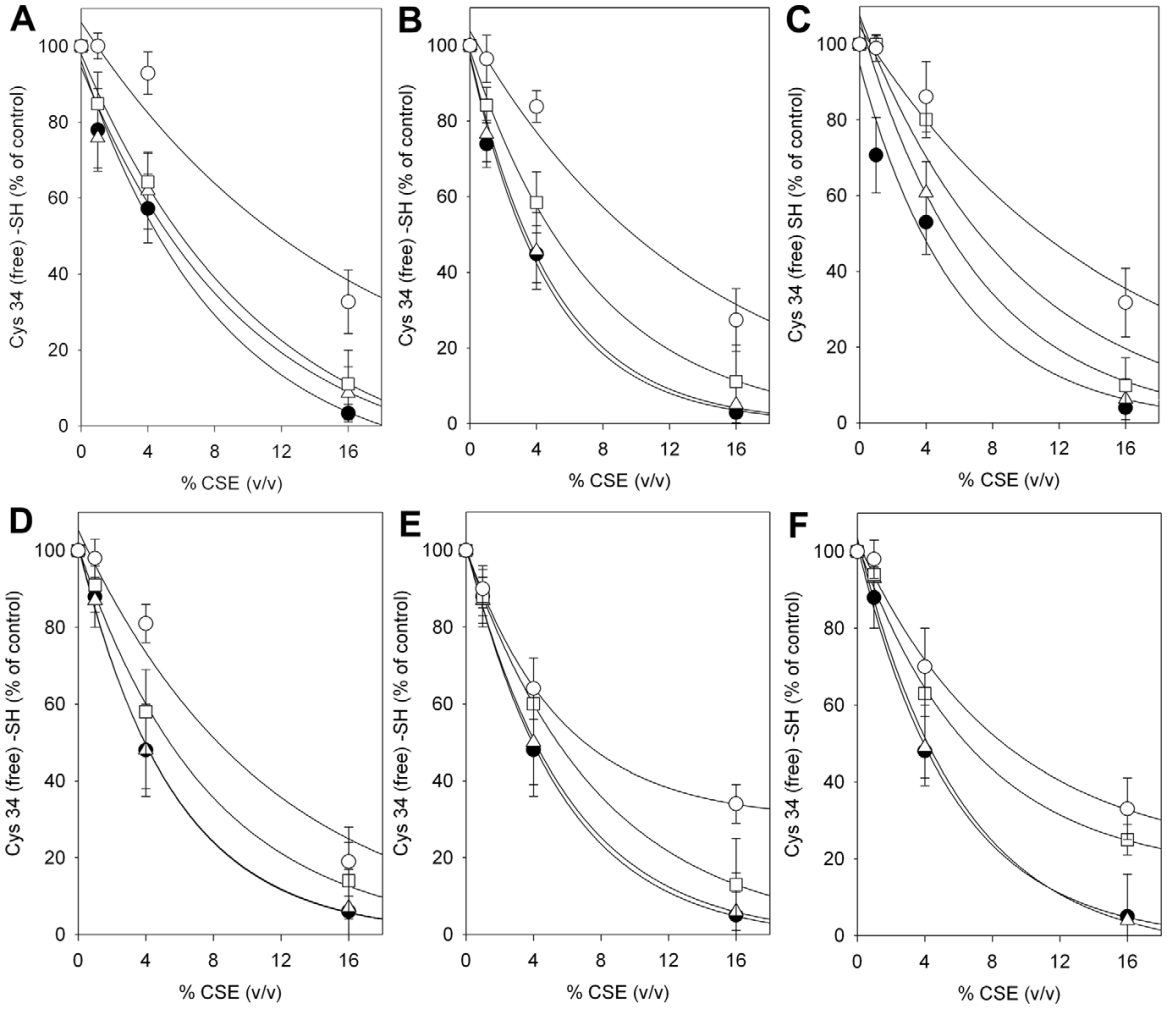
Effect of GSH, Cys-SH, or NAC on CSE-induced Cys34 oxidation. (**A**) HSA-SH solutions (60 µM) were incubated with vehicle (control; filled circles) or 0.3 µM (triangles), 3 µM (squares) or 30 µM (open circles) GSH (molar ratio [GSH]/[HSA] = 0.005–0.05–0.5, respectively), for 30 min, before exposing protein solutions to 0%, 1%, 4% and 16% (v/v) CSE, for 60 min. (**B**) HSA-SH solutions (60 µM) were incubated with vehicle (control; filled circles) or 1 µM (triangles), 10 µM (squares) or 100 µM (open circles) Cys-SH (molar ratio [Cys-SH]/[HSA] = 0.016–0.16–1.6, respectively), for 30 min, before exposing protein solutions to 0%, 1%, 4% and 16% (v/v) CSE, for 60 min. (**C**) HSA-SH solutions (60 µM) were incubated with vehicle (control; filled circles) or 1 µM (triangles), 10 µM (squares) or 100 µM (open circles) NAC (molar ratio [NAC]/[HSA] = 0.016–0.16–1.6, respectively), for 30 min, before exposing protein solutions to 0%, 1%, 4% and 16% (v/v) CSE, for 60 min. (**D**) Experiment was performed as in (**A**) except that HSA solutions were exposed to CSE immediately after GSH addition. (**E**) Experiment was performed as in (**B**) except that HSA solutions were exposed to CSE immediately after Cys-SH addition. (**F**) Experiment was performed as in (**C**) except that HSA solutions were exposed to CSE immediately after NAC addition. Conditions of incubation and estimation of HSA Cys34 free sulfhydryl group by biotin–HPDP binding and Western blot analysis are described under Materials and methods. Densitometric analyses of biotin-HPDP incorporation are shown. Data are presented as the mean ± SD of three independent determinations.

Plasma low molecular mass aminothiols such as GSH and Cys-SH could potentially provide some protection against CSE-induced oxidation of HSA Cys34 thiol group. Human plasma concentrations of GSH and Cys-SH are in the range 2–5 µM and 8–10 µM, respectively [8], [11], [38]. Therefore, the possibility of preventing CSE-induced oxidation of the Cys34 sulfhydryl group was investigated by incubating HSA-SH solutions with increasing concentrations of GSH (Fig. 3A) or Cys-SH (Fig. 3B) for 30 min, before exposing protein samples to 1–16% (v/v) CSE. Concentrations of GSH and Cys-SH were chosen so as to reproduce the mean physiological blood molar ratios of aminothiols to HSA as well as molar ratios of one and two orders of magnitude higher. Physiological concentrations (relative to that of HSA) of both GSH (molar ratio [GSH]/[HSA] = 0.005, where [GSH] = 0.3 µM) (Fig. 3A, triangles) and Cys-SH (molar ratio [Cys-SH]/[HSA] = 0.016, where [Cys-SH] = 1 µM) (Fig. 3B, triangles) produced no protection. Physiological plasma concentrations of other antioxidants including ascorbic acid, methionine, and uric acid were also ineffective (data not shown). In contrast, both GSH and Cys-SH partially prevented CSE-induced oxidation of the Cys34 thiol at supra-physiological concentrations relative to that of HSA (molar ratio [GSH]/[HSA] = 0.05–0.5, where [GSH] = 3–30 µM, and molar ratio [Cys-SH]/[HSA] = 0.16–1.6, where [Cys-SH] = 10–100 µM). Supra-physiological amounts (3–30 µM) of GSH produced, respectively, minimal (less than 10%) or moderate (about 30%) protection against oxidation of the Cys34 thiol induced by CSE (Fig. 3A). Supra-physiological (relative to that of HSA) concentrations of Cys-SH, namely, 10 µM and 100 µM, produced a slightly higher protection of about 10 and 30–40%, respectively (Fig. 3B). The highest concentration of both aminothiols afforded the greatest protection towards HSA exposed to 1–4% CSE.

As a 30-min pre-incubation with the thiols could promote auto-oxidation, in particular of Cys, we performed analogous experiments with parallel samples in which GSH or Cys-SH were added just before exposure to CSE, obtaining results similar to those obtained after pre-incubation with the thiols (Fig. 3D,E).

### Effects of pharmacological NAC concentrations on CSE-induced Cys34 oxidation

The potential protective effect of NAC was also considered. The diverse pharmacological applications of NAC are inherent in the multifaceted chemical properties of its constituent, cysteinyl thiol, which enable NAC to act as a nucleophile as well as a scavenger of ROS [39]. A series of HSA-SH samples were incubated with 1, 10 or 100 µM NAC (concentrations that equate to those found in plasma after tolerable oral NAC dosing (∼800 mg/m^2^/day) and enhancing intracellular GSH levels, while maintaining a low side-effect profile [40]) for 30 min before exposure to 1–16% (v/v) CSE in order to investigate the possibility of preventing CSE-induced oxidation of Cys34 sulfhydryl group by means of NAC supplementation. Fig. 3C shows that the lowest concentration of NAC (1 µM) produced negligible protection (about 5%) of the HSA thiol. Higher NAC concentrations (10–100 µM) induced higher protection, reducing oxidation of Cys34 –SH group by about 20 and 35%, respectively (Fig. 3C).

An eventual NAC auto-oxidation occurring during the 30-min pre-incubation was checked performing analogous experiments with parallel HSA-SH samples in which NAC was added just before exposure to CSE. The obtained results were similar to those obtained after pre-incubation with NAC (Fig. 3F).

### Prevention of CSE-induced Cys34 oxidation by means of supra-physiological concentrations of GSH, Cys-SH, or NAC

**Figure 4:**
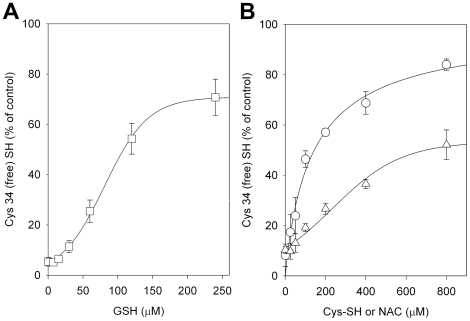
Prevention of CSE-induced oxidation of HSA Cys34 sulfhydryl group by means of supra-physiological concentrations of GSH, Cys-SH, or NAC. HSA-SH solutions (60 µM) were incubated for 30 min with different concentrations of (**A**) GSH (squares), (**B**) Cys-SH (triangles) or NAC (open circles) so as to obtain molar ratios of each aminothiol to HSA-SH equal to 25∶1, 50∶1, 100∶1, 200∶1, 400∶1, 800∶1 compared with the physiological plasma ratio, before exposing protein solutions to 16% (v/v) CSE, for 60 min. Results are shown as concentration of the Cys34 sulfhydryl group. Estimation of HSA Cys34 free sulfhydryl group by biotin–HPDP binding and Western blot analysis are described under Materials and methods. Densitometric analysis of biotin-HPDP incorporation is shown. Data are presented as the mean ± SD of three independent determinations.

All the three antioxidants strongly suppressed oxidation of the sulfhydryl group of Cys34 elicited by HSA exposure to 16% CSE only at exaggeratedly high concentrations of each aminothiol, i.e., at molar ratios of aminothiols to HSA of hundreds-fold (i.e., 10×, 25×, 50×, 100×, 200×, 400×, 800×) compared with the physiological plasma ratios (Fig. 4). For example, a 50% protection against CSE-induced oxidation of Cys34 thiol group was afforded by 120 µM GSH, corresponding to a [GSH]/[HSA] molar ratio = 2 (i.e., 400×).

### Human RBCs protect the Cys34 sulfhydryl group of HSA against CSE oxidizing effects

**Figure 5:**
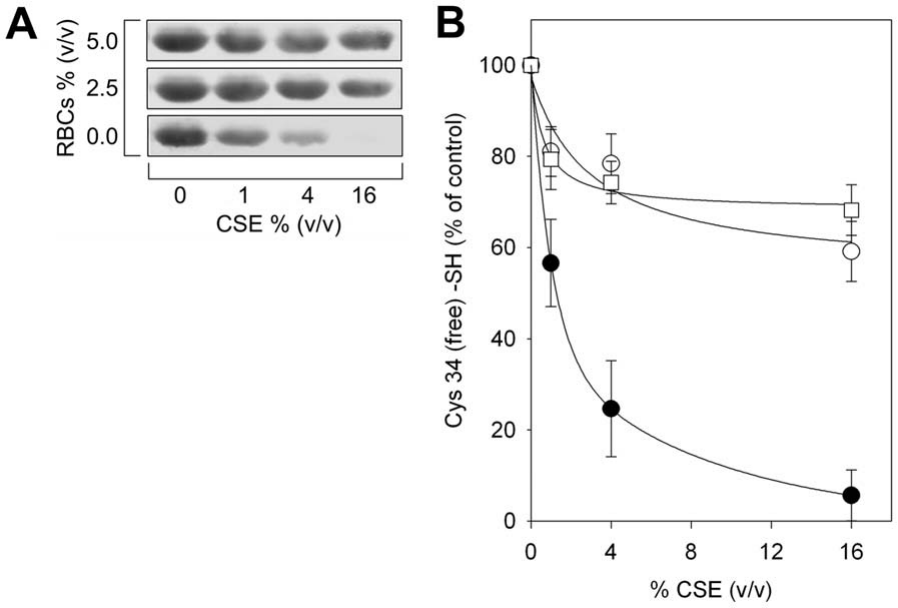
RBCs protect the Cys34 sulfhydryl group of HSA against CSE oxidizing effects. HSA-SH solutions (60 µM) were exposed to 1–16% (v/v) CSE in the absence (filled circles) or presence of 2.5% (v/v; open circles) or 5% (v/v; open squares) erythrocytes in 200 mM PBS/NaCl, containing 5 mM glucose. After a 60-min incubation at 37°C, samples were centrifuged and HSA solutions were recovered from the supernatants. (**A**) The extent of CSE-induced oxidation of HSA Cys34 free sulfhydryl group was then evaluated by biotin-HPDP, as described under Materials and methods, followed by Western blot analysis with streptavidin-HRP and enhanced chemiluminescence, after protein separation by non-reducing SDS-PAGE. (**B**) Graph shows densitometric analysis of the HSA protein band, corresponding to biotin-HPDP incorporation. Data are presented as the mean ± SD of three independent determinations.

Previous studies carried out to determine the occurrence of oxidative modifications in plasma proteins of smokers did not specifically evidence HSA oxidation, indeed the contrary, as fibrinogen was the only oxidized (carbonylated) protein found in human plasma of smokers [21]. Consequently, also considering the preponderance of RBCs in blood as well as their high GSH content, we asked whether human RBCs were protective against CSE-induced thiol oxidation of HSA Cys34. HSA-SH solutions were exposed to 1–16% (v/v) CSE in the absence or presence of 2.5% (v/v) or 5% (v/v) erythrocytes. After a 60-min incubation, samples were centrifuged and HSA solutions were recovered from the supernatants. The extent of CSE-induced oxidation of HSA Cys34 free sulfhydryl group was then evaluated by tagging –SH groups with biotin-HPDP, followed by Western blot analysis with streptavidin-HRP and enhanced chemiluminescence, after protein separation by non-reducing SDS-PAGE (Fig. 5). As shown in Fig. 5A, HSA exposed to CSE in the presence of RBCs clearly exhibited an increase in the reduced form of Cys34 as compared with the protein alone exposed to CSE. The related graph shows densitometric analysis of biotin-HPDP incorporation (Fig. 5B).

**Figure 6:**
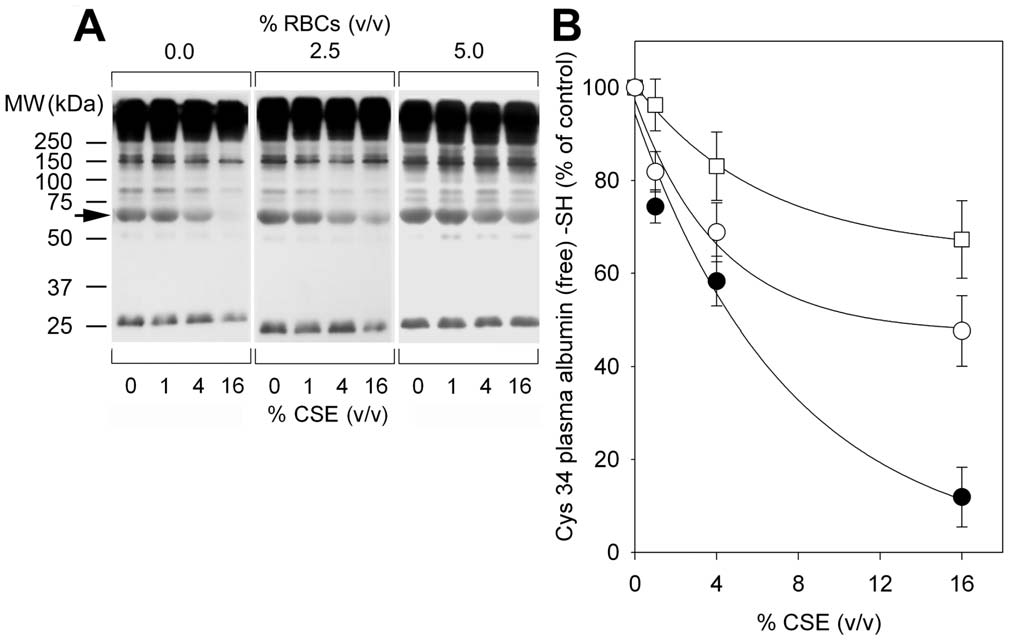
RBCs protect plasma protein sulfhydryl groups against CSE oxidizing effects. Human plasma diluted 1∶10 (i.e., at the protein concentration of 4 mg/ml) with PBS/NaCl was incubated with 1–16% (v/v) CSE in the absence (filled circles) or presence of 2.5% (v/v; open circles) or 5% (v/v; open squares) erythrocytes. After a 60-min incubation at 37°C, samples were centrifuged and protein solutions were recovered from the supernatants. (**A**) The extent of CSE-induced oxidation of plasma protein free sulfhydryl groups was then evaluated by biotin-HPDP, as described under Materials and methods, followed by Western blot analysis with streptavidin-HRP and enhanced chemiluminescence, after protein separation by non-reducing SDS-PAGE. Arrow indicates the HSA protein band. Molecular weight (MW) markers are indicated on the left. (**B**) Graph shows densitometric analysis of the HSA protein band, corresponding to biotin-HPDP incorporation in HSA Cys34.

**Figure 7:**
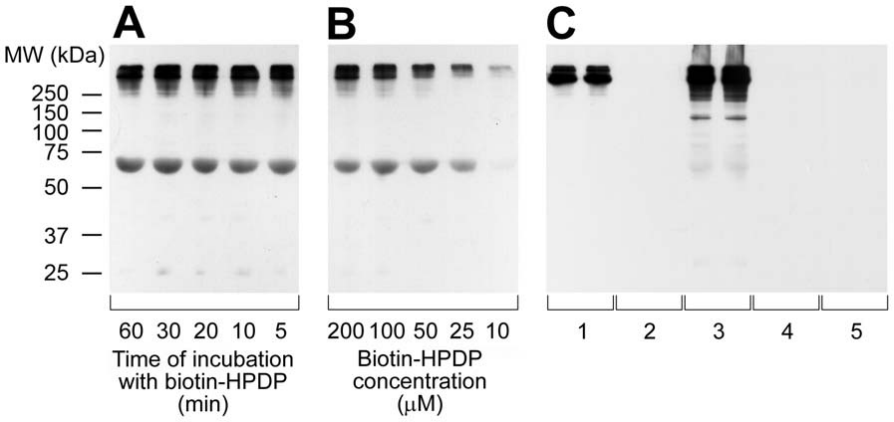
Estimation of a possible non-specific biotin-HPDP binding to human plasma proteins. All the experiments were performed with control human plasma proteins, i.e. not exposed to CSE, in the absence of RBCs. (**A**) Human plasma was diluted at the protein concentration of 4 mg/ml with PBS/NaCl and the extent of free sulfhydryl groups was evaluated by biotin-HPDP, varying the time of incubation with biotin-HPDP between 5 and 60 min. All the other experimental conditions for the biotinylation reaction, non-reducing SDS-PAGE, Western blot analysis with streptavidin-HRP and enhanced chemiluminescence were the same as those described under Materials and methods. Molecular weight (MW) markers are indicated on the left. (**B**) Human plasma was diluted at the protein concentration of 4 mg/ml with PBS/NaCl and the extent of free sulfhydryl groups was evaluated by biotin-HPDP, varying its concentration between 10 and 200 µM. All the other experimental conditions for the biotinylation reaction, non-reducing SDS-PAGE, Western blot analysis with streptavidin-HRP and enhanced chemiluminescence were the same as those described under Materials and methods. (**C**) Human plasma was diluted at the protein concentration of 4 mg/ml with PBS/NaCl. Plasma proteins were incubated for 30 min at 50°C with 20 mM NEM, precipitated with 100% acetone and protein pellets were resuspended with PBS/NaCl containing 0.4 mM biotin–HPDP (for further details, see Materials and Methods) (lanes 1); plasma proteins were incubated with 0.4 mM biotin instead of biotin-HPDP (the biotinylation reaction was performed as described under Materials and Methods) (lanes 2); plasma proteins were incubated for 30 min at 50°C with 20 mM NEM before starting the biotinylation reaction with 0.4 mM biotin–HPDP (for further details, see Materials and Methods) (lanes 3); plasma proteins were incubated for 30 min at 50°C with 20 mM NEM but were not subject to the biotinylation reaction (lanes 4); plasma proteins were not subject to the biotinylation reaction (lanes 5). In panels A and B, each electrophoretic lane was loaded with one-half of the amount of total proteins loaded in panel C and in Fig. 6A. After non-reducing SDS-PAGE, the Western blot was developed as described under Materials and Methods.

The protective effect of RBCs against CSE-induced HSA Cys34 thiol oxidation was also observed when an analogous experiment was carried out with human plasma proteins instead of the only HSA (Fig. 6). Human plasma diluted 1∶10 with PBS/NaCl was incubated with 1–16% (v/v) CSE in the absence or presence of 2.5% (v/v) or 5% (v/v) erythrocytes. After a 60-min incubation at 37°C, samples were processed as described above. The results of the biotin-based tagging experiment, after separation by non-reducing SDS-PAGE and Western blotting with HRP-streptavidin, are presented in Fig. 6A. Each band represents a protein containing one or more reduced or reversibly oxidized protein thiol. Increasing band intensity indicates increasing amount of reduced protein thiols due to the concurrent presence of RBCs in the incubation mixture with CSE. This is clearly evident for HSA (arrow) as well as for a few other plasma proteins. The related graph shows densitometric analysis of biotin-HPDP incorporation in HSA Cys34 (Fig. 6B). However, Fig. 6A also shows very strong signals of higher molecular weight than albumin. This is in apparent conflict with albumin being the most abundant (∼80%) thiol in human plasma [16]–[18]. Therefore, we performed a series of additional experiments aimed at explaining such an apparent contradiction (Fig. 7). As the very strong signals of higher molecular weight than albumin shown in Fig. 6A could be due to a non-specific binding of biotin-HPDP, we repeated the experiment with control plasma proteins in the absence of RBCs, diluting human plasma to 1∶10 with PBS/NaCl and varying either the time of incubation with biotin-HPDP (Fig. 7A) or its concentration (Fig. 7B), whereas all the other experimental conditions were the same as those of the experiment shown in Fig. 6. In both cases, the chemiluminescence signal from proteins of higher molecular weight than albumin remained markedly higher than that from HSA (Fig. 7A,B). Finally, we performed a series of experiments with or without NEM in order to verify the eventuality of a non-specific binding of biotin-HPDP to sites other than protein sulfhydryl groups (Fig. 7C). In particular, plasma proteins (4 mg/ml, i.e., human plasma was diluted 1∶10 with PBS/NaCl) were incubated for 30 min at 50°C with 20 mM NEM, precipitated with three volumes of 100% acetone and protein pellets were resuspended with PBS/NaCl containing 0.4 mM biotin–HPDP (for further details, see Materials and Methods) (Fig. 7C, lanes 1); plasma proteins (4 mg/ml) were incubated with 0.4 mM biotin instead of biotin-HPDP (the biotinylation reaction was performed as described under Materials and Methods) (Fig. 7C, lanes 2); plasma proteins (4 mg/ml) were incubated for 30 min at 50°C with 20 mM NEM before starting the biotinylation reaction with 0.4 mM biotin–HPDP (for further details, see Materials and Methods) (Fig. 7C, lanes 3); plasma proteins (4 mg/ml) were incubated for 30 min at 50°C with 20 mM NEM but were not subject to the biotinylation reaction (Fig. 7C, lanes 4); plasma proteins (4 mg/ml) were not subject to the biotinylation reaction (Fig. 7C, lanes 5). After non-reducing SDS-PAGE, the Western blot was developed as described under Materials and Methods. The chemiluminescence signal clearly evident in plasma protein samples alkylated with NEM, which covalently binds to sulfhydryl groups, and further biotinylated (Fig. 7C, lanes 1 and 3) suggests that biotin-HPDH binds to some plasma proteins in a non-specific way, i.e., non dependent on the HPDP mojety interaction with –SH groups.

### Human RBCs protect human plasma proteins against CSE-induced protein carbonylation

**Figure 8:**
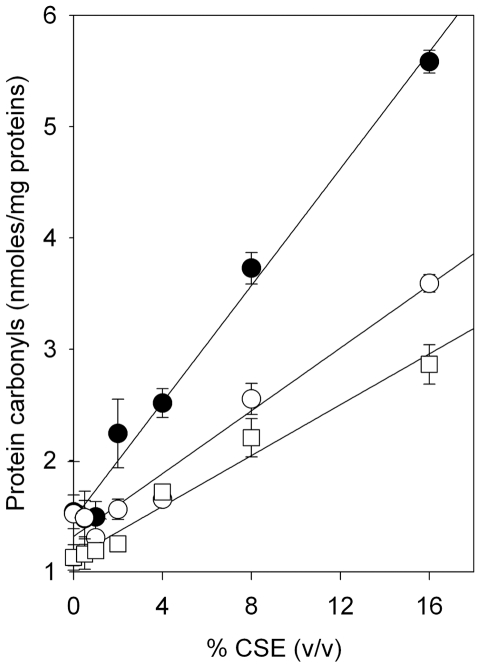
RBCs protect plasma proteins against CSE-induced carbonylation. Human plasma diluted 1∶10 (i.e., at the protein concentration of 4 mg/ml) with PBS/NaCl was incubated with 1–16% (v/v) CSE in the absence (filled circles) or presence of 2.5% (v/v; open circles) or 5% (v/v; open squares) erythrocytes. After a 60-min incubation at 37°C, samples were centrifuged and protein solutions were recovered from the supernatants. The extent of CSE-induced carbonylation of plasma proteins was then evaluated by spectrophotometric determination after protein carbonyl derivatization with DNPH, as described under Materials and methods. Data are presented as the mean ± SD of three independent determinations.

To determine whether erythrocytes protected human plasma proteins also against CSE-induced carbonylation, human plasma was diluted 1∶10 with PBS/NaCl and protein solutions were exposed to 1–16% (v/v) CSE in the absence or presence of 2.5% (v/v) or 5% (v/v) erythrocytes (i.e., equal to about one tenth of the mean human hematocrit value as in our experiments plasma protein concentration too is equal to about one tenth of the mean *in vivo* concentration) in PBS/NaCl containing 5 mM glucose. After a 60-min incubation at 37°C, samples were centrifuged at 10,000 *g* for 20 s, at room temperature and plasma proteins recovered from the supernatants were processed for determining the extent of protein carbonylation as described in the Materials and methods section. Fig. 8 shows that human RBCs effectively protect plasma proteins from CSE-induced protein carbonylation.

## Discussion

In plasma of healthy young adults, 70–80% of total HSA contains the fully reduced sulfhydryl group of Cys34; ∼25% of the Cys34 forms a mixed disulfide with low molecular mass aminothiols, generating *S*-thiolated albumin; a small fraction (2–5%) of albumin Cys34 is more highly oxidized to the sulfinic (HSA-SO_2_H) or sulfonic acid (HSA-SO_3_H) form, which cannot usually be reversed with DTT and can cause loss of protein function [13], [41], [42]. Albumin antioxidant role is mostly attributed to its sole cysteine thiol, that of Cys34, which accounts for the bulk of free thiols in plasma [15], and its ability to scavenge various oxidizing species [13], [17], [41], [43]. Although HSA Cys34 does not react particularly fast with oxidants, HSA-SH can be considered an important plasma scavenger and a key element of antioxidant defenses due to its very high concentration [13], [17], [18], [41]–[45]. The oxidized forms of albumin are picked up in the circulation as they are not present in albumin secreted from the liver cells [15].

The purpose of the present study was twofold. Firstly, because some low molecular mass plasma antioxidants have been proposed to have a protective effect against exposure to CS [46], we examined the protective effects of some plasma antioxidants and the synthetic aminothiol NAC against CSE-induced Cys34 oxidation in vitro. Secondly, given the high GSH concentration (∼3 mM) within erythrocytes as well as the great number of RBCs in human blood, where no oxidized form of HSA has been detected in smokers [20]–[23], we asked whether RBCs were protective towards CSE-induced oxidation of both HSA and total human plasma proteins.

When HSA-SH solutions were incubated with increasing concentrations (0–16% v/v) of CSE, the Cys34 thiol pool progressively decreased, as determined by both DTNB assay (Fig. 1) and biotin-HPDP binding revealed by Western blotting analysis (Fig. 1, inset). The observed diminution in the level of Cys34 –SH groups was not ascribable to formation of intermolecular disulfide bridges. Thiol oxidation induced by exposure of HSA-SH to 1–16% CSE was essentially reversible, as judged by the almost complete recovery of biotin-HPDP binding when CSE-treated albumin samples were either further incubated in the presence of 1 mM DTT (though a negligible amount of the biotinylation signal may be due to the opening of a few intramolecular disulfides; see lane 2) (Fig. 2A) or dialyzed against PBS added with 5 mM GSH (Fig. 2A, strips c, e, g, l plus GSH), except for a minimal amount (about 5–6%) of, likely, irreversible oxidations occurring in HSA samples treated with 16% CSE (Fig. 2B). Therefore, we can guess that the great majority of oxidative modifications occurring at the Cys34 –SH group are reversible ones.

The possibility of preventing CSE-induced oxidation of albumin Cys34 by physiological low molecular mass aminothiols was investigated by incubating HSA-SH with increasing concentrations of GSH or Cys-SH before protein exposure to CSE. Our results suggest that plasma concentrations of GSH and Cys-SH are ineffective in preventing CS–induced oxidation of HSA Cys34 (Fig. 3). Differently, the redox status of the Cys34 thiol group is partially preserved by incubation with GSH (Fig. 3A) or Cys-SH (Fig. 3B) at supra-physiological concentrations before exposure to CSE. The synthetic antioxidant NAC, whose use includes the treatment of a variety of diseases sharing alterations of the redox status and GSH depletion as well as dietary supplementation, is an analogue and precursor of L–cysteine and GSH, which exhibits the ability to scavenge ROS and could significantly increase the GSH level in the plasma [39]. NAC at the lowest concentration produced very limited protection against CSE-induced Cys34 oxidation, whereas the antioxidant preventive effect against HSA thiol oxidation was more pronounced when using higher concentrations of NAC (Fig. 3C). At the highest CSE concentration (16% v/v), the strong oxidation of the Cys34 sulfhydryl group was partially prevented only in the presence of extremely high (not physiological) concentrations of GSH, Cys-SH or NAC, the latter exerting the most effective antioxidant effect (Fig. 4). A marked protective effect against the CSE-induced oxidation of HSA Cys34 thiol was exerted by human RBCs, whose “antioxidant” action was effective not only when HSA alone was exposed to CSE (Fig. 5) but also when total human plasma proteins were exposed to the oxidizing insult (Fig. 6). In addition, the RBC “antioxidant” action was not limited to protecting protein thiols (Figs. 5 and 6) but resulted to be effective also in protecting total plasma proteins from CSE-induced carbonylation (Fig. 8).

This study suggests the possibility that GSH, Cys-SH, and NAC at exceedingly supra-physiological concentrations could provide some benefit in preventing or reducing oxidative modifications of HSA Cys34 in smokers, also considering that smoking alters plasma thiol homeostasis, causing an oxidation in both the plasma GSH/GSSG redox and the Cys-SH/cystine redox and significantly decreasing the plasma cysteine pool size [10]. However, it seems highly improbable that such exaggerate concentrations of those aminothiols may be reached in human plasma by means of nutritional supplementation and/or pharmacological interventions designed to improve plasma thiol homeostasis in smokers. In addition, a recent study involving 156 daily smokers showed that participants who believed that they were taking a dietary supplement smoked more cigarettes than did controls. This study suggests that smokers' use of dietary supplements may create illusory invulnerability, which, in turn, reduces the self-regulation of smoking [47].

By contrast, our results provide evidence that plasma (physiological) concentrations of GSH and Cys-SH were absolutely ineffective as well as other antioxidants, i.e., ascorbic acid, methionine, and uric acid, which are strong reducing agents and potent antioxidants that act together in circulation, at plasma (physiological) concentrations were similarly ineffective to prevent the CSE–induced thiol oxidation and carbonylation of HSA. As a whole, the role of antioxidants in preventing smoke-associated diseases remains controversial [48]–[54].

Several studies have shown that RBCs are important as biological carriers of GSH, the major antioxidant in erythrocytes, where its concentration is approximately 3 mM, by *de novo* synthesis and as such appear to provide an important detoxifying system within the circulation [55], [56]. This could at least in part explain why oxidized/carbonylated HSA has been found within extravascular fluids (e.g., in the brochoalveolar lavage fluid) and parenchymal lung tissue in cigarette smokers [24]–[26] but not within blood circulation [20]–[23]. This could also partly justify the protective action shown by RBCs against CSE-induced oxidation of both HSA and total plasma proteins (Figures 5, 6, and 8), although such a protective effect cannot be due only to GSH, as suggested by the only ∼50% protection shown by GSH at low millimolar concentrations, i.e., at a concentration eight hundred-fold compared with the physiological plasma ratio and comparable to that found within RBCs (Figure 4). It can be hypothesized that the antioxidant protection afforded by RBCs against CSE-induced oxidation may also be due to their other rich antioxidant systems [57].

In erythrocytes the major antioxidant is GSH, which protects important proteins such as spectrin, the oxidation of which can lead to increased membrane stiffness. GSH not only supports antioxidant defense, but is also an important sulfhydryl buffer, maintaining –SH groups in hemoglobin and enzymes in the reduced state [58]. Compared with other somatic cells, erythrocytes are exposed to oxidative stress from a wide variety of sources; therefore, RBCs are well equipped with many antioxidants, besides GSH. Despite their lack of mitochondria, as well as that of nucleus, ribosomes, and every other intracellular organelle, which are lost when the RBC emerges from the bone marrow, ROS are continuously produced within human RBCs due to the high O_2_ tension in arterial blood and their abundant heme Fe content within the O_2_-carrier hemoglobin, which is able to initiate a wide array of free radical reactions [59]. Erythrocytes transport large amount of O_2_ and nitric oxide (NO^•^) over their lifespan resulting in oxidative stress [60], [61]. Various factors can lead to the generation of oxidizing species in erythrocytes: evidence indicates that many physiological and pathological conditions such as aging, inflammation, eryptosis (apoptosis-like process of RBCs, also known as erythroptosis) develop through ROS and/or RNS action. A major source of ROS in erythrocytes is hemoglobin, which undergoes auto-oxidation to produce O_2_^•−^. Since the intraerythrocytic concentration of oxygenated hemoglobin is 5 mM, even a small rate of auto-oxidation can produce substantial levels of ROS [62]. As a consequence, RBCs have potent antioxidant protection consisting of both enzymatic and non-enzymatic systems (the latter not limited to GSH) that modify highly ROS/RNS into substantially less reactive intermediates [57], [63]. Under physiological conditions, RBCs exert a scavenging activity towards ROS/RNS often over-produced in morbidity states, e.g., in inflamed tissues, or when they experience hyperglycemic conditions after a meal [63]. Indeed, RBCs are equipped with a very efficient antioxidant machinery that ensures a reducing environment to maintain both a functional spectrin-based skeleton-membrane interaction and hemoglobin in a Fe^2+^-active form. Thus, under physiological conditions, RBCs serve the important function of circulating “scrubbers”: as ROS/RNS scavenging “devices”, RBCs can improve organism's antioxidant defenses.

In detail, human erythrocytes are well equipped with non-enzymatic antioxidants such as GSH, thioredoxin, vitamin C, and vitamin E. Furthermore, compared with other cell types, RBCs exhibit high activities of the most important antioxidant enzymes, including superoxide dismutase, thioredoxin/thioredoxin reductase system, peroxiredoxin, catalase, glutathione peroxidase, glutathione reductase, plasma membrane oxidoreductases, to reduce extracellular oxidants and, finally, the methemoglobin reductase/NADH/glycolysis system to maintain hemoglobin in a Fe^2+^-active form [63].

Peroxiredoxins (Prxs) constitute a family of ubiquitous thiol-dependent, homodimeric peroxidases that reduce hydrogen peroxide (H_2_O_2_) and alkyl hydroperoxides to water and alcohol, respectively. They rely on a conserved Cys residue to catalyze peroxide reduction [64]. The catalytic cycle involves the reduction of oxidized Prx by thioredoxin and reduction capacity of NADPH via NADPH -thioredoxin reductase [65], [66]. When Prx2 (a typical 2-Cys Prx) reacts with peroxide, the peroxidatic cysteine at the active site on one subunit is oxidized to a sulfenic acid. A second conserved cysteine at the C-terminal end of the other subunit (the resolving cysteine) then reacts with the sulfenic acid to form a disulfide bridge. Reduction of the disulfide by thioredoxin regenerates Prx2 and completes the cycle. Thioredoxin is in turn regenerated by thioredoxin reductase, with reducing equivalents derived from NADPH [64]. An intriguing feature of mammalian 2-Cys Prxs is that, in the presence of high levels of peroxide, the peroxidatic Cys becomes overoxidized to the sulfinic or sulfonic acid form [67]. This abolishes the enzyme's peroxidase activity, although overoxidized Prx can be slowly reverted to the reduced state by sulfiredoxin or sestrins [68], [69]. It has been suggested that overoxidation allows intracellular accumulation of H_2_O_2_, which can then function as a signal transducer for various pathways [70].

Erythrocyte Prx2 is the third most abundant erythrocyte protein (∼250 µM in the cytosol, equivalent to ∼15 million copies per cell) [31] after hemoglobin and carbonic anhydrase and reacts with peroxides with rate constants six-eight orders of magnitude faster than GSH [71]–[73]. Erythrocytes also possess Prx1, Prx3 and Prx6, although in lesser amounts than Prx2. Erythrocyte Prxs also contribute as peroxynitrite scavengers in the circulation, being also able to act as peroxynitrite reductases catalytically very efficiently [74]. Prx2 is able to protect hemoglobin from exogenous oxidation [75], and is thought to remove hydroperoxides at the erythrocyte membrane [76]. In erythrocytes, Prx2 is peculiarly resistant to overoxidation (i.e., oxidation of the cysteine thiol to a sulfinic/sulfonic acid). Furthermore, erythrocyte Prx2 is extremely efficient at scavenging H_2_O_2_ non-catalytically and competes effectively with catalase and glutathione peroxidase to scavenge low levels of H_2_O_2_: it is remarkably sensitive to reversible oxidation by H_2_O_2_ concentrations in the low micromolar range. However, recycling of the oxidized dimer occurs very slowly [71], [73]. Although it does not act as a classical antioxidant enzyme, its high concentration and substrate sensitivity enable it to handle low H_2_O_2_ concentrations efficiently without the need for recycling. This large excess of Prx2 over its substrate suggests that Prx2 does not function in the erythrocyte as a classical erythrocyte antioxidant enzyme, but as a very effective H_2_O_2_ scavenging protein [71], [73]. These unique redox properties may account for its non-redundant role in erythrocyte defense against oxidative stress.

Altogether this powerful antioxidant machinery makes the RBC a highly efficient antioxidant system not yet fully appreciated. Moreover, specialized mechanisms have been evolved to repair and eventually remove damaged proteins as well as damaged lipids. RBCs, in fact, being devoid of protein synthesis, must be equipped with several mechanisms, not yet completely clarified, to counteract cell alterations induced by ROS/RNS or, alternatively, to signal irreversibly damaged cells to the reticulo-endothelial system for their removal [77].

In conclusion, given the importance of cigarette smoking as a risk factor for numerous diseases and the pathophysiological role played by oxidative stress in these illnesses, quitting smoking represents an irreplaceable preventive strategy against CS-induced oxidative stress and oxidative damage. Smoking cessation is followed by symptom improvement and by a marked increase in plasma concentrations of vitamins A, C, E, uric acid, total thiols, and carotenoids, and substantially improves plasma resistance towards oxidative challenge [78], although the oxidant burden in the airways continues for months [79]. However, endeavours in identifying new and more efficacious antioxidants as a therapeutic strategy should continue. Under this point of view, it could be useful to study in detail the specific RBC antioxidant systems exerting protection against CS-induced oxidative damage to plasma proteins, in order to verify the possibility of potentiating them in smokers. The mobility of RBCs makes them an antioxidant not only for their local environment, but also an oxidant scavenger throughout the circulation [57]. Erythrocytes' efficient intracellular antioxidant machinery, coupled with their high blood concentration, renders RBCs an effective “sink” of reactive species [80]. Indeed, RBCs can act as scavengers for plasma hydrogen peroxide and superoxide anion [81] as well as of nitric oxide radical in circulation, because of their high concentration (∼9 mM) of hemoglobin [57]. Therefore, the scavenging ability of RBCs could benefit not only the blood per se, but more importantly, the entire organism. Thus, researchers planning to investigate the effect of CS on the blood redox status could take into account the biological peculiarities of RBCs.
